# P2X7 Receptor Activation Induces Reactive Oxygen Species Formation and Cell Death in Murine EOC13 Microglia

**DOI:** 10.1155/2013/271813

**Published:** 2013-01-27

**Authors:** Rachael Bartlett, Justin J. Yerbury, Ronald Sluyter

**Affiliations:** ^1^School of Biological Sciences, University of Wollongong, Wollongong, NSW 2522, Australia; ^2^Illawarra Health and Medical Research Institute, Wollongong, NSW 2522, Australia

## Abstract

The P2X7 purinergic receptor is a ligand-gated cation channel expressed on leukocytes including microglia. This study aimed to determine if P2X7 activation induces the uptake of organic cations, reactive oxygen species (ROS) formation, and death in the murine microglial EOC13 cell line. Using the murine macrophage J774 cell line as a positive control, RT-PCR, immunoblotting, and immunolabelling established the presence of P2X7 in EOC13 cells. A cytofluorometric assay demonstrated that the P2X7 agonists adenosine-5′-triphosphate (ATP) and 2′(3′)-O-(4-benzoylbenzoyl) ATP induced ethidium^+^ or YO-PRO-1^2+^ uptake into both cell lines. ATP induced ethidium^+^ uptake into EOC13 cells in a concentration-dependent manner, with an EC_50_ of *~*130 **μ**M. The P2X7 antagonists Brilliant Blue G, A438079, AZ10606120, and AZ11645373 inhibited ATP-induced cation uptake into EOC13 cells by 75–100%. A cytofluorometric assay demonstrated that P2X7 activation induced ROS formation in EOC13 cells, via a mechanism independent of Ca^2+^ influx and K^+^ efflux. Cytofluorometric measurements of Annexin-V binding and 7AAD uptake demonstrated that P2X7 activation induced EOC13 cell death. The ROS scavenger N-acetyl-L-cysteine impaired both P2X7-induced EOC13 ROS formation and cell death, suggesting that ROS mediate P2X7-induced EOC13 death. In conclusion, P2X7 activation induces the uptake of organic cations, ROS formation, and death in EOC13 microglia.

## 1. Introduction

Microglia are the resident innate immune cells of the central nervous system (CNS) and play an important role in immune surveillance [1] and in the pathogenesis and progression of a number of CNS disorders [2]. Microglia are constantly mobile, spending time scanning the extracellular space of the CNS [1]. In response to brain injury or immunological stimuli, these cells become activated and undergo dramatic morphological and functional changes, which are highly dependent on the context of their activation [3]. Activated microglia phagocytose debris and peptides, present antigens, and produce a number of soluble factors. These factors may be inflammatory, regulatory, or cytotoxic in nature and include reactive oxygen species (ROS), nitric oxide (NO), proinflammatory and anti-inflammatory cytokines, prostaglandins, and growth factors [4, 5]. Microglia can be both neuroprotective or neurotoxic when activated, depending on the factors they produce and the quantity and context in which they are released, with prolonged or excessive activation of these cells being associated with neuroinflammation and the progression of a number of CNS disorders [6]. The mechanisms behind enhanced microglial activation in these disorders and the features determining the balance between neuroprotection and neurotoxicity are not fully understood.

The P2X7 receptor is a trimeric ligand-gated cation channel belonging to the P2X family of purinergic receptors [7]. P2X7 is predominately expressed on mononuclear leukocytes including macrophages and microglia and plays a role in inflammation and immunity [8]. In particular, P2X7 is currently receiving attention due to its possible roles in neuroinflammation [9]. Activation of P2X7 by extracellular adenosine-5′-triphosphate (ATP), or the most potent P2X7 agonist, 2′(3′)-O-(4-benzoylbenzoyl) ATP (BzATP), causes the passage of small cations including Ca^2+^, Na^+^, and K^+^ across the plasma membrane, as well as organic cations, such as the fluorescent dyes ethidium^+^ and YO-PRO-1^2+^ [7]. Compared to other P2X receptors, P2X7 requires relatively high ATP concentrations for activation, with a half maximal effective concentration (EC_50_) of 100–300 *μ*M [7]. Activation of P2X7 leads to a number of cell-specific downstream signalling events, including the formation of ROS and reactive nitrogen species [10], and either cell proliferation or death [11, 12].

Using molecular, immunochemical, and pharmacological techniques, we demonstrate in the current study that the murine microglial EOC13 cell line [13] expresses functional P2X7. Activation of P2X7 by ATP in this cell line induces the uptake of organic cations, ROS formation, and cell death.

## 2. Materials and Methods

### 2.1. Reagents and Antibodies

RPMI-1640 and DMEM/F12 media, GlutaMAX, normal horse serum (NHS), 0.05% trypsin, YO-PRO-1^2+^, 2′,7′-dichlorodihydrofluorescein diacetate (H_2_DCFDA), and 2′,7′-difluorofluorescein diacetate (DAF-FM DA) were from Invitrogen (Grand Island, NY). Fetal bovine serum (FBS) (heat-inactivated before use) was from Bovogen Biologicals (East Keilor, Australia). ATP, BzATP, ethidium bromide, dimethyl sulfoxide (DMSO), glycerol gelatin, and the P2X7 antagonist Brilliant Blue G (BBG) were from Sigma-Aldrich (St. Louis, MO). The P2X7 antagonists A438079, AZ10606120, and AZ11645373 were from Tocris Bioscience (Ellisville, MO). Primers were from GeneWorks (Hindmarsh, Australia). Protease inhibitor cocktail tablets (complete, Mini, EDTA-free) and Annexin-V-Fluorescein were from Roche Diagnostics (Penzberg, Germany). SuperSignal West Pico Chemiluminescent Substrate was from Pierce (Rockford, IL). The viability dye 7-aminoactinomycin D (7AAD) and the ROS scavenger N-acetyl-L-cysteine (NAC) were from Enzo Life Sciences (Plymouth Meeting, PA). The broad-spectrum ROS inhibitor diphenyleneiodonium (DPI) was from Cayman Chemical (Ann Arbor, MI). Phenyl-methyl-sulfonyl-fluoride (PMSF), n-dodecyl *β*-D-maltoside, and ethylene glycol tetraacetic acid (EGTA) were from Amresco (Solon, OH).

Cells preincubated with H_2_O soluble compounds (BBG, A438079, and AZ10606120) were compared to cells preincubated in the absence of each compound. Cells preincubated with DMSO soluble compounds (AZ11645373 and DPI) were compared to cells preincubated with DMSO alone. Solutions containing 40 mM NAC were prepared in NaCl medium (140 mM NaCl, 5 mM NaOH, 5 mM KCl, 10 mM HEPES, and 5 mM glucose, pH 7.4) and adjusted to pH 7.4; cells were then preincubated in NaCl medium with or without NAC. When studied, antagonists/inhibitors were present during incubations with ATP.

Rabbit anti-mouse P2X7 (extracellular epitope) polyclonal antibody (Ab) and rabbit anti-rat P2X7 (C-termini epitope) Ab (and corresponding blocking peptide) were from Alomone Labs (Jerusalem, Israel). Peroxidase-conjugated goat anti-rabbit IgG Ab was from Rockland Immunochemicals (Gilbertsville, PA). Cy3-conjugated donkey anti-rabbit IgG Ab was from Jackson ImmunoResearch (West Grove, PA). Rat anti-mouse P2X7 monoclonal antibody (mAb) (clone HANO43) was from Enzo Life Sciences. Rat IgG2b isotype control mAb and allophycocyanin- (APC-) conjugated donkey anti-rat IgG Ab were from eBioscience (San Diego, CA).

### 2.2. Cell Lines

The murine macrophage J774 cell line, the murine microglial EOC13 cell line, and the murine lymphoblast LADMAC cell line, all originally obtained from the American Type Culture Collection (Manassas, VA), were kindly provided by Jasmyn Dunn (University of Queensland, Brisbane, Australia) (J774) and Iain Campbell (University of Sydney, Sydney, Australia) (EOC13 and LADMAC). J774 cells were maintained in RPMI-1640 medium containing 10% FBS and 2 mM GlutaMAX (complete RPMI medium). EOC13 cells were maintained in DMEM/F12 supplemented with 10% FBS, 2 mM GlutaMAX, and 20% LADMAC conditioned medium (complete DMEM medium). Cell lines were maintained at 37°C and 95% air/5% CO_2_ and passaged every 3-4 days. Quarterly mycoplasma testing was carried out using the MycoAlert Mycoplasma Detection Kit (Lonza, Rockland, ME), as per the manufacturer's instructions. For experiments, cells were harvested by cell scraping unless otherwise stated.

### 2.3. Fluorescent Cation Dye Uptake Assay

Cells were washed in NaCl medium (300 ×*g* for 5 min), resuspended in NaCl medium, and equilibrated at 37°C for 5 min (1 × 10^5^ cells/1 mL/tube). Cells were then incubated with 25 *μ*M ethidium^+^ (or 1 *μ*M YO-PRO-1^2+^ where indicated) in the absence or presence of the P2X7 agonists ATP or BzATP (as indicated) for 5 min. In some experiments, ATP-induced cation uptake was assessed with cells suspended in KCl medium (150 mM KCl, 5 mM glucose, and 10 mM HEPES, pH 7.4) or in NaCl medium containing 1 mM CaCl_2_ or 100 *μ*M EGTA. In other experiments, cells were preincubated in the absence or presence of P2X7 antagonists or the ROS scavenger NAC (as indicated) for 15 and 30 min, respectively, prior to cation and ATP addition. Incubations with nucleotides were stopped by the addition of an equal volume of ice-cold NaCl medium containing 20 mM MgCl_2_ (MgCl_2_ medium) followed by centrifugation (300 ×*g* for 5 min). Cells were washed once with NaCl medium and events collected using a LSR II flow cytometer (BD Biosciences, San Diego, CA) (excitation 488 nm, emission collected with 575/26 and 515/20 band-pass filters for ethidium^+^ and YO-PRO-1^2+^, resp.). The mean fluorescence intensity (MFI) of relative cation uptake was determined using FlowJo software (Tree Star, Ashland, OR).

### 2.4. P2X7 Expression by RT-PCR

Total RNA isolation from cells was performed using the RNeasy Mini Kit (Qiagen, Hilden, Germany) as per the manufacturer's instructions. PCR amplification was performed as described previously [14] using SuperScript III One-Step RT-PCR System Platinum Taq DNA polymerase (Invitrogen) with 500 ng of RNA, and P2X7 forward (5′-ATATCCACTTCCCCGGCCAC-3′) and reverse (5′-TCGGCAGTGATGGGACCAG-3′) primers for 42 cycles (94°C, 1 min; 68°C, 1 min; 72°C, 1 min). PCR products were separated on a 2% agarose gel in Tris-acetate EDTA buffer and visualised with ethidium bromide staining. Images of gels were collected using a Gel Logic 212 PRO imaging system (Carestream Health, Rochester, NY).

### 2.5. P2X7 Protein Detection by Immunoblotting

Cells were washed three times with phosphate-buffered saline (PBS) (300 ×*g* for 5 min) and lysed (1 × 10^7^ cells/mL) over 60 min in ice-cold lysis buffer (50 mM BisTris, 750 mM 6-aminohexanoic acid, 1% n-dodecyl *β*-D-maltoside, 1 mM PMSF, and protease inhibitor cocktail, pH 7.0). Cells were sheared by passing ten times through a 21 G needle and stored at −20°C until needed. Cells were then thawed and cleared (16,000 ×*g* at 4°C for 10 min). Supernatants (25 *μ*g protein/lane) were separated under reducing conditions (5%  *β*-mercaptoethanol) using a discontinuous SDS-PAGE system with a 4% stacking gel and 10% separating gel. Proteins were then transferred to nitrocellulose membranes (Bio-Rad, Hercules, CA) and blocked at 4°C overnight with Tris-buffered saline (250 mM NaCl and 50 mM Tris, pH 7.5) containing 0.2% Tween-20 and 5% milk powder. The following day, nitrocellulose membranes were incubated at room temperature for 2 h with anti-mouse P2X7 Ab (1 : 500) in Tris-buffered saline containing 0.2% Tween-20 and 5% milk powder. Membranes were washed three times over 30 min with Tris-buffered saline containing 0.2% Tween-20 and then incubated at room temperature for 1 h with peroxidise-conjugated anti-IgG Ab (1 : 1000) in Tris-buffered saline containing 0.2% Tween-20 and 5% milk powder. Membranes were washed as above and visualised using chemiluminescent substrate and Amersham Hyperfilm ECL (GE Healthcare, Little Chalfont, Buckinghamshire, UK). Images of films were collected using a GS-800 Calibrated Densitometer (Bio-Rad).

### 2.6. Cell Surface P2X7 Protein Detection by Flow Cytometry

Cells in NaCl medium containing 10% NHS and 0.02% NaN_3_ (1 × 10^5^ cells/200 *μ*L/tube) were incubated with anti-P2X7 or rat IgG2b isotype control mAb (5 *μ*g/mL) at room temperature for 30 min. Cells were then washed twice with NaCl medium (300 ×*g* for 5 min) and incubated with APC-conjugated anti-rat IgG Ab (1.3 *μ*g/mL) and 7AAD (to exclude dead cells) for 30 min protected from light. Cells were washed once as above. Events were then collected using a LSR II flow cytometer (excitation 633 nm, emission collected with 660/20 band-pass filter for APC; excitation 488 nm, emission collected with 695/40 band-pass filter for 7AAD). Relative cell-surface P2X7 was determined using FlowJo software and is expressed as the difference in the MFI of specific mAb labelling and isotype control labelling.

### 2.7. P2X7 Protein Detection by Confocal Microscopy

EOC13 or J774 cells in their respective complete culture medium were plated into 24-well plates with 13 mm glass coverslips (5 × 10^4^ cells/0.5 mL/well) and incubated at 37°C, 95% air/5% CO_2_ overnight to allow time to adhere. The following day, cells were fixed with 4% paraformaldehyde in PBS at room temperature for 15 min and then washed three times with PBS over 10 min. Cells were incubated with permeabilisation solution (PBS containing 0.1% DMSO, 2% NHS, and 0.1% Triton X-100) at room temperature for 10 min and washed three times with PBS. Cells were then blocked with 20% NHS in PBS at room temperature for 20 min. Cells were incubated at 4°C overnight with anti-rat P2X7 Ab (5 *μ*g/mL; preincubated for 1 h in the absence or presence of blocking peptide as per the manufacturer's instruction) in PBS containing 1% BSA, 0.2% NHS, and 0.05% NaN_3_. Cells were then washed as above and incubated at room temperature for 1 h with Cy3-conjugated anti-rabbit IgG Ab (15 *μ*g/mL) in PBS containing 0.2% NHS. Cells were washed as above and then the coverslips mounted onto slides with 50% (v/v) glycerol gelatin in PBS. Coverslips were sealed with nail varnish. Cells were visualised using a DM IBRE inverted microscope and TCS SP confocal imaging system (Leica, Mannheim, Germany) (excitation 488 nm, emission collected at 560–600 nm). Images were captured using Leica Confocal Software.

### 2.8. ROS Formation Assay

EOC13 cells in complete DMEM medium were plated into 24-well plates (5 × 10^4^ cells/0.5 mL/well) and incubated at 37°C, 95% air/5% CO_2_ overnight. Cells were then incubated with NaCl medium containing 10 *μ*M H_2_DCFDA (0.5 mL/well) at 37°C, 95% air/5% CO_2_, protected from light for 30 min. The medium was removed, and cells were further incubated in NaCl medium (containing 1 mM CaCl_2_) in the absence or presence of 2 mM ATP at 37°C, 95% air/5% CO_2_ for 15 min. Incubations were stopped by the addition of an equal volume of ice-cold MgCl_2_ medium. Cells were harvested using 0.05% trypsin (5 min, 37°C) and were washed once with NaCl medium. Events were collected using a LSR II flow cytometer (excitation 488 nm, emission collected at 515/20 nm) and the MFI of relative dichlorofluorescein (DCF) determined using FlowJo software.

In some experiments, ATP-induced ROS formation was assessed in KCl medium, in NaCl medium in the absence of 1 mM CaCl_2_ or presence of 100 *μ*M EGTA, or in complete DMEM medium in the absence or presence of 10 *μ*M AZ10606120 (15 min preincubation, prior to ATP addition). As free Ca^2+^ lowers the concentration of ATP^4−^ [15], cells incubated in the absence of 1 mM Ca^2+^ were incubated with 1.4 mM ATP to provide equimolar ATP^4−^ concentrations (575 *μ*M), as calculated using the Bound and Determined Program [16]. In other experiments, cells were preincubated in the absence or presence of AZ10606120, NAC, or DPI (as indicated) for 15, 30, and 30 min, respectively, prior to ATP addition. Cells prior to harvesting were also visualised by differential interference contrast (DIC) imaging using an Eclipse TE2000 inverted microscope (Nikon, Tokyo, Japan) to examine cell morphology, and DIC images were captured using Image-Pro AMS (Version 6.1) (Media Cybernetics, Rockville, MD).

### 2.9. NO Formation Assay

EOC13 cells in complete DMEM medium were plated into 24-well plates (5 × 10^4^ cells/0.5 mL/well) and incubated at 37°C, 95% air/5% CO_2_ overnight. Cells were then incubated with NaCl medium containing 10 *μ*M DAF-FM DA (0.5 mL/well) at 37°C, 95% air/5% CO_2_, protected from light for 30 min. The medium was removed, and the cells were washed once. Cells were then preincubated with NaCl medium in the absence or presence of 10 *μ*M AZ10606120 at 37°C, 95% air/5% CO_2_ for 15 min. Following this, cells were further incubated in the absence or presence of 1.4 mM ATP for 15 min. Incubations were stopped by the addition of an equal volume of ice-cold MgCl_2_ medium. Cells were harvested using 0.05% trypsin (5 min, 37°C) and were washed once with NaCl medium. Events were collected using a LSR II flow cytometer (excitation 488 nm, emission collected at 515/20 nm) and the MFI of relative benzotriazole derivative determined using FlowJo software.

### 2.10. Cell Death Assay

EOC13 cells in complete DMEM medium were plated into 24-well plates (5 × 10^4^ cells/0.5 mL/well) and incubated at 37°C, 95% air/5% CO_2_ overnight. Cells were then incubated with filter sterile ATP (as indicated) at 37°C, 5% CO_2_ for 24 h. In some experiments, cells were preincubated in the absence or presence of 10 *μ*M AZ10606120 or 40 mM NAC for 15 or 30 min, respectively, prior to ATP addition. In other experiments, cells were preincubated in the absence or presence of 40 mM NAC for 90 min, with 2 mM ATP added in the final 15–60 min, and then the medium replaced and cells incubated at 37°C, 95% air/5% CO_2_ for a further 24 h. Following the 24 h incubations, cells were harvested from wells using 0.05% trypsin (5 min, 37°C) and washed once with Annexin-V binding medium (NaCl medium containing 5 mM CaCl_2_). Cells were then incubated with Annexin-V binding medium containing Annexin-V-Fluorescein and 7AAD at room temperature protected from light for 15 min. Events were collected using a LSR II flow cytometer (excitation 488 nm, emission collected with 515/20 and 695/40 band-pass filters for Annexin-V-Fluorescein and 7AAD, resp.) and the MFI of Annexin-V-Fluorescein and 7AAD determined using FlowJo software. Quadrant markers were used to determine the percentage of Annexin-V^+^/7AAD^−^, Annexin-V^−^/7AAD^+^, and Annexin-V^+^/7AAD^+^ cells. In some experiments, cells prior to harvesting were visualised by DIC imaging to examine cell morphology, and DIC images captured as outlined in Section  2.8.

### 2.11. Data Presentation and Statistical Analyses

Data is presented as the mean ± SD. Differences between multiple treatments were compared by ANOVA paired with Tukey's HSD posttest using Prism 5 for Windows (Version 5.04) (GraphPad Software, San Diego, CA), with differences considered significant for *P* < 0.05. Concentration response and inhibition curves were fitted using Prism 5 and assuming a variable slope, with normalised and nonnormalised response curves, respectively, selected to obtain the best fit. Estimates of EC_50_ values and half maximal inhibitory concentrations (IC_50_) were obtained from individual fits of these plots.

## 3. Results

### 3.1. P2X7 Antagonists Inhibit ATP-Induced Ethidium^+^ Uptake into J774 Macrophage Cells in a Concentration-Dependent Manner

**Figure 1:**
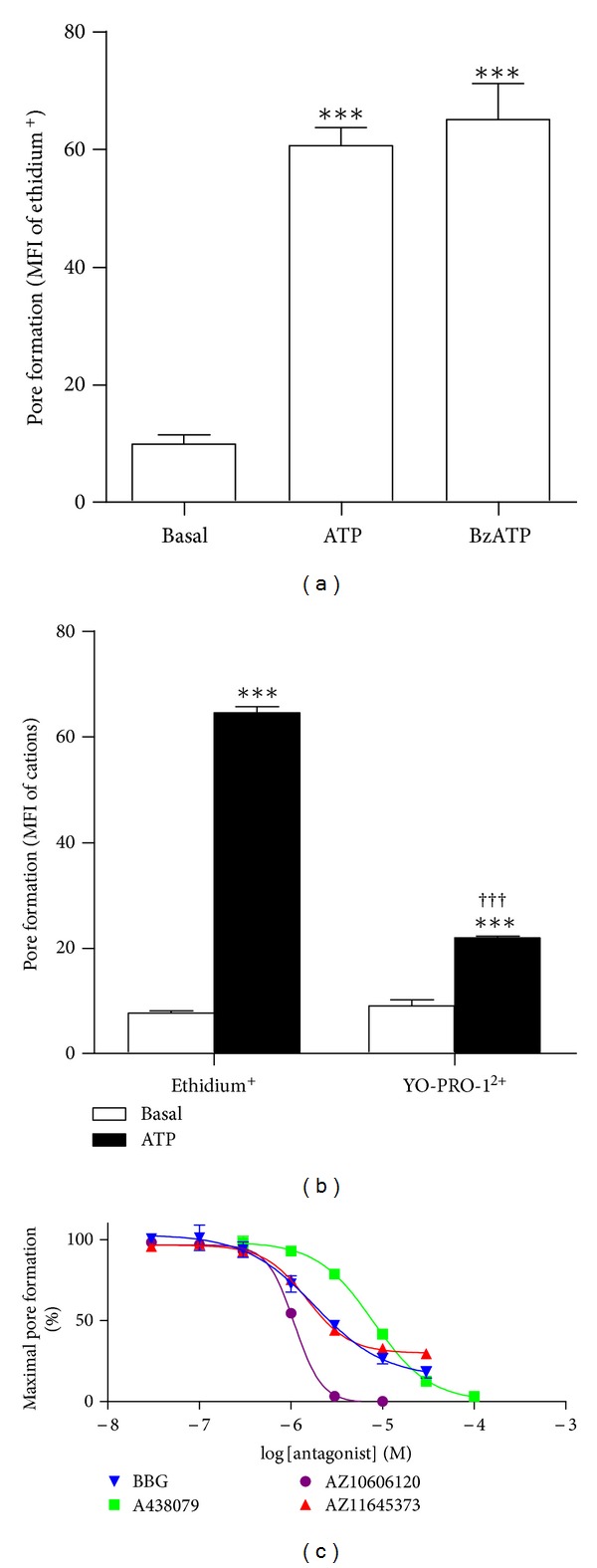
P2X7 antagonists inhibit ATP-induced ethidium^+^ uptake into J774 macrophage cells in a concentration-dependent manner. (a and b) J774 cells in NaCl medium were incubated with (a and b) 25 *μ*M ethidium^+^ or (b) 1 *μ*M YO-PRO-1^2+^ in the absence (basal) or presence of (a and b) 1 mM ATP or (a) 0.1 mM BzATP at 37°C for 5 min. (c) Cells in NaCl medium were preincubated with Brilliant Blue G (BBG), A438079, AZ10606120, and AZ11645373 (as indicated) at 37°C for 15 min. Ethidium^+^ (25 *μ*M) was then added, and cells were incubated in the absence or presence of 1 mM ATP at 37°C for 5 min. (a–c) Incubations were stopped by the addition of MgCl_2_ medium and centrifugation. Mean fluorescence intensity (MFI) of fluorescent cation uptake (pore formation) was determined by flow cytometry. (a and b) Results shown as means ± SD, *n* = 3; ***P < 0.001 compared to corresponding basal; ^†††^ *P* < 0.001 compared to corresponding ATP. (c) Curves presented as a percentage of the maximal ATP-induced ethidium^+^ uptake and expressed as the mean ± SD, *n* = 3-4.

The murine macrophage J774 cell line is well known to express functional P2X7 [17]. Moreover, our group has demonstrated the presence of functional P2X7 in various cell types using a fixed-time fluorescent cation uptake assay (e.g., [14, 18]). Therefore, this technique was used to confirm the presence of P2X7 in J774 cells and to validate the use of this cell line as a positive control. Incubation of J774 cells with the P2X7 agonist ATP and the most potent P2X7 agonist BzATP induced significant ethidium^+^ uptake into these cells compared to cells incubated in the absence of nucleotide (Figure 1(a)). In addition, incubation of J774 cells with ATP induced significant YO-PRO-1^2+^ uptake compared to cells incubated in the absence of ATP (Figure 1(b)). However, ATP-induced YO-PRO-1^2+^ uptake was significantly lower than ATP-induced ethidium^+^ uptake (Figure 1(b)).

A number of highly specific P2X7 antagonists, including A438079 [19], AZ10606120 [20], and AZ11645373 [21], have recently become available. In addition, BBG is commonly used as a P2X7 antagonist *in vitro* and *in vivo* (e.g., [22, 23]). Therefore, to determine the optimum concentrations of these antagonists required to inhibit murine P2X7, J774 cells were preincubated in the absence or presence of varying concentrations of BBG, A438079, AZ10606120, and AZ11645373 and the ATP-induced ethidium^+^ uptake assessed. Each antagonist impaired 1 mM ATP-induced ethidium^+^ uptake in a concentration-dependent manner, with IC_50_ values of 1.8 ± 0.2, 7.9 ± 0.4, 1.0 ± 0.1, and 1.5 ± 0.1 *μ*M, respectively (Figure 1(c)). AZ10606120 and A438079 completely inhibited ethidium^+^ uptake at respective concentrations of 10 and 100 *μ*M. In contrast, BBG and AZ11645373 were partial antagonists at the ATP concentration used (1 mM).

### 3.2. EOC13 Microglial Cells Express P2X7

**Figure 2:**
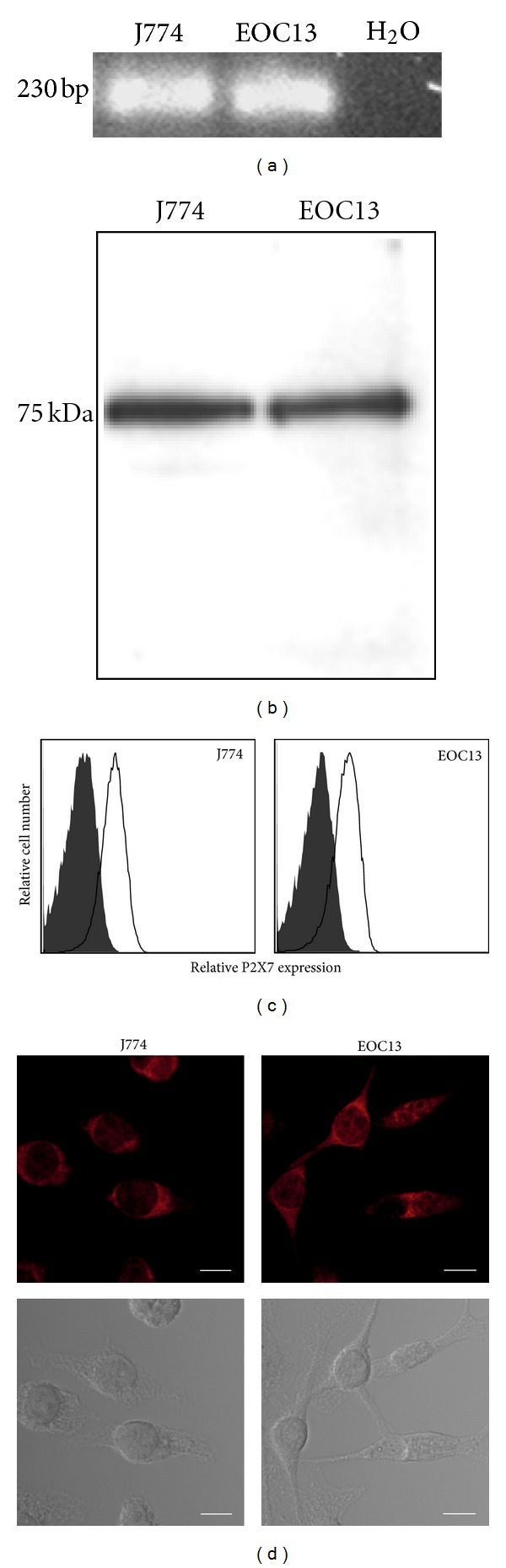
EOC13 microglial cells express P2X7. (a) RNA from EOC13 and J774 cells was amplified by RT-PCR using primers for P2X7. Water in place of RNA was included as a negative control in the PCR reaction. PCR products were separated and visualised with ethidium bromide staining. (b) EOC13 and J774 cell lysates were separated by SDS-PAGE, transferred to nitrocellulose, and probed with an anti-P2X7 Ab. (c) EOC13 and J774 cells were labelled with an anti-P2X7 (solid line) or isotype control (shaded) mAb and then with APC-conjugated anti-IgG Ab and 7AAD (to exclude dead cells). Relative P2X7 expression (mean fluorescence intensity) was determined by flow cytometry. (d) Fixed and permeabilised EOC13 and J774 cells were labelled with an anti-P2X7 Ab and then with Cy3-conjugated anti-IgG Ab. P2X7 (top panels) and phase contrast (bottom panels) images were assessed by confocal microscopy. Bars represent 10 *μ*m. (a–d) Results are representative of 2-3 experiments.

To determine whether EOC13 microglial cells express P2X7, a series of experiments using J774 cells as a positive control were performed. Firstly, RNA was isolated from EOC13 and J774 cells and amplified by RT-PCR using primers for P2X7. Similar to J774 cells, EOC13 cells were found to express P2X7 mRNA, as evident from the 230 base pair band corresponding to the size of the predicted product (Figure 2(a)). The presence of total P2X7 protein was determined by probing separated whole lysates of both cell lines with an anti-P2X7 Ab. Immunoblotting revealed one major protein band of 75 kDa for both EOC13 and J774 cells (Figure 2(b)), corresponding to the predicted size of glycosylated P2X7. Moreover, both cell lines were incubated with an anti-P2X7 mAb and the presence of cell-surface P2X7 determined by flow cytometry. Immunolabelling demonstrated cell-surface P2X7 on both EOC13 and J774 cells, with MFIs of 13 ± 2 and 14 ± 4, respectively (*n* = 3) (Figure 2(c)). Finally, both cell lines were stained with an anti-P2X7 Ab and analysed by confocal microscopy. This similarly demonstrated the presence of cell-surface P2X7, as well as intracellular P2X7, with bright staining observed on all cells (Figure 2(d)). Preincubation of the anti-P2X7 Ab with blocking peptide completely abrogated the detection of P2X7 in both cell lines (data not shown). Together, these results indicate that P2X7 is expressed in EOC13 cells.

### 3.3. EOC13 Microglial Cells Express Functional P2X7

**Figure 3:**
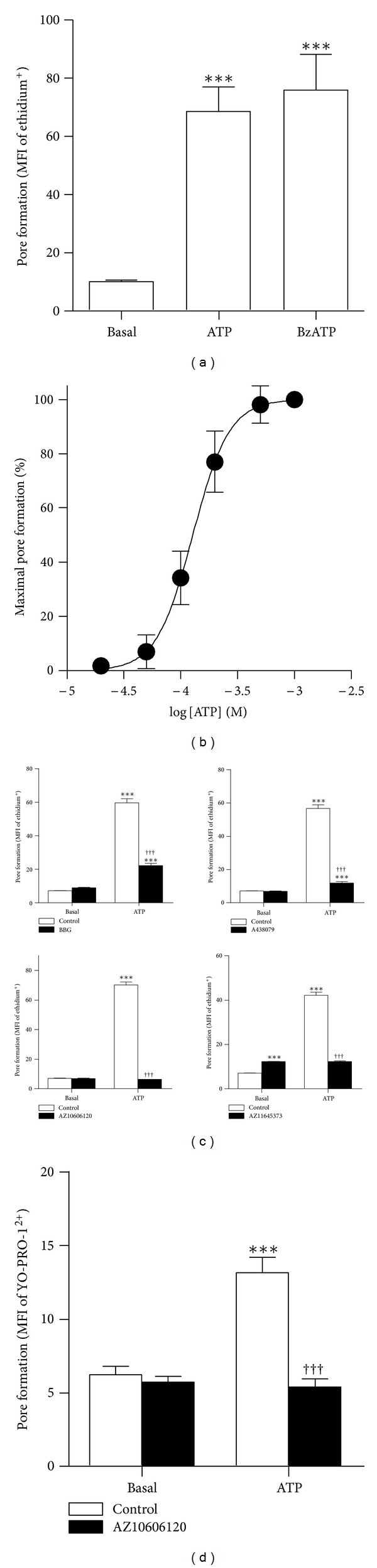
EOC13 microglial cells express functional P2X7. (a and b) EOC13 cells in NaCl medium were incubated with 25 *μ*M ethidium^+^ in the absence (basal) or presence of (a) 1 mM ATP, 0.1 mM BzATP, or (b) varying concentrations of ATP (as indicated) at 37°C for 5 min. (c and d) Cells in NaCl medium were preincubated in the absence (control) or presence of (c) 30 *μ*M Brilliant Blue G (BBG), 100 *μ*M A438079, 30 *μ*M AZ11645373, or (c and d) 10 *μ*M AZ10606120 at 37°C for 15 min. (c) Ethidium^+^ (25 *μ*M) or (d) YO-PRO-1^2+^ (1 *μ*M) was then added, and (c and d) cells were incubated in the absence (basal) or presence of 1 mM ATP at 37°C for 5 min. (a–d) Incubations were stopped by the addition of MgCl_2_ medium and centrifugation. Mean fluorescence intensity (MFI) of fluorescent cation uptake (pore formation) was determined by flow cytometry. (a, c, and d) Results shown as means ± SD, *n* = 3; ***P < 0.001 compared to corresponding basal; ^†††^ *P* < 0.001 compared to corresponding ATP in the absence of antagonist. (b) Curve presented as a percentage of the maximal ATP-induced ethidium^+^ uptake and expressed as the mean ± SD, *n* = 3.

To determine whether P2X7 was functional in EOC13 cells, the fixed-time ethidium^+^ uptake assay was performed. Both ATP and BzATP were found to induce significant ethidium^+^ uptake into EOC13 cells compared to cells incubated in the absence of nucleotide (Figure 3(a)). Next, EOC13 cells were incubated with increasing concentrations of ATP. ATP induced ethidium^+^ uptake in a concentration-dependent manner, with maximal uptake obtained at an ATP concentration of 1 mM and with an EC_50_ of 130 ± 30 *μ*M (Figure 3(b)). Subsequent characterisations of P2X7 in EOC13 microglia were performed using this maximal concentration of ATP (1 mM).

To determine if the observed ATP-induced ethidium^+^ uptake into EOC13 cells was mediated by P2X7, cells were preincubated in the absence or presence of P2X7 antagonists at inhibitory concentrations optimal for 1 mM ATP-induced ethidium^+^ uptake in J774 cells, as demonstrated above (Figure 1(c)). Preincubation of EOC13 cells with 30 *μ*M BBG, 100 *μ*M A438079, 10 *μ*M AZ10606120, and 30 *μ*M AZ11645373 resulted in significant impairment of ATP-induced ethidium^+^ uptake by 75 ± 2, 90 ± 1, 100 ± 0, and 99 ± 1%, respectively (Figure 3(c)). None of the P2X7 antagonists except AZ11645373 significantly altered the basal ethidium^+^ uptake into EOC13 cells. Again with the exception of AZ11645373, which reduced the amount of gated viable cells by ~30%, cell viability (as assessed by forward and side scatter) was similar between treatments (data not shown).

To determine if P2X7 activation could induce the uptake of a second organic cation into EOC13 cells, cells were preincubated in the absence or presence of AZ10606120, and ATP-induced YO-PRO-1^2+^ uptake examined. Similar to ethidium^+^ uptake, 1 mM ATP induced significant YO-PRO-1^2+^ uptake into EOC13 cells compared to cells incubated in the absence of ATP (Figure 3(d)). Moreover, preincubation of cells with 10 *μ*M AZ10606120 resulted in complete inhibition of ATP-induced YO-PRO-1^2+^ uptake (Figure 3(d)). Incubation with AZ10606120 did not significantly alter the basal YO-PRO-1^2+^ uptake (Figure 3(d)). Furthermore, cell viability (as assessed by forward and side scatter) was similar between treatments (data not shown). Collectively, these results indicate that P2X7 is functional in EOC13 cells.

### 3.4. P2X7 Activation Induces ROS Formation in EOC13 Microglial Cells

**Figure 4:**
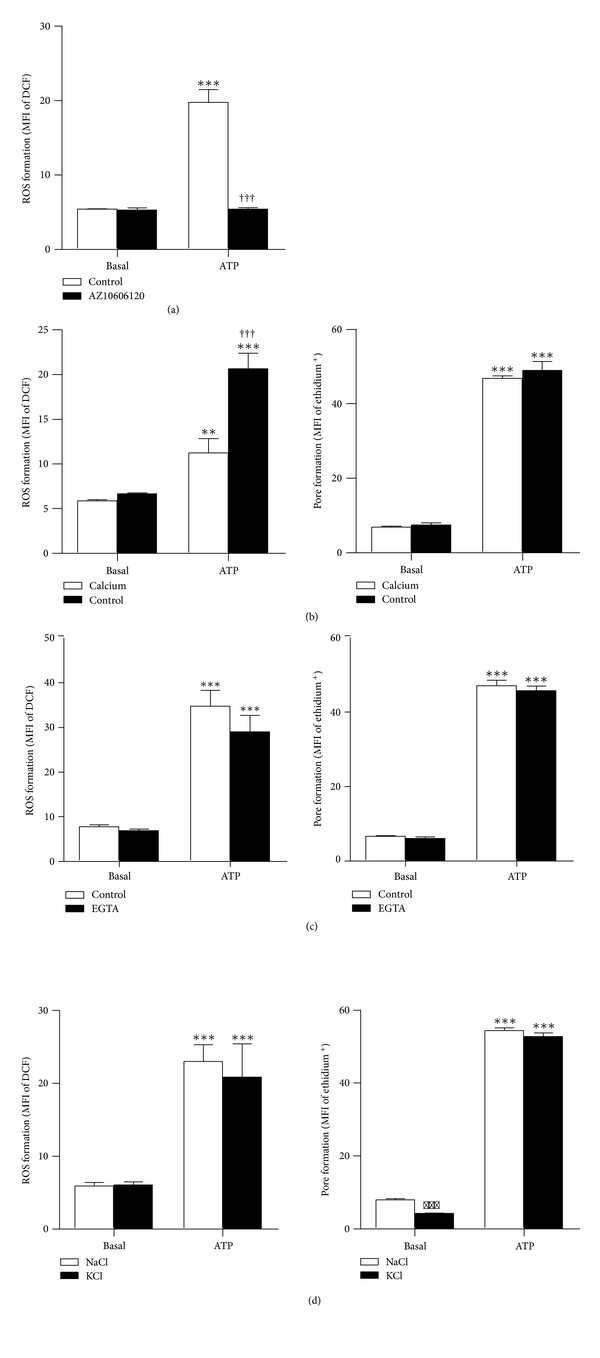
P2X7 activation induces ROS formation in EOC13 microglial cells. (Left panels) Adherent DCF-loaded EOC13 cells or (right panels) suspended EOC13 cells in (a) NaCl medium containing 1 mM Ca^2+^ (preincubated in the absence (control) or presence of 10 *μ*M AZ10606120 at 37°C for 15 min), (b) NaCl medium in the absence (control) or presence of 1 mM Ca^2+^, (c) NaCl medium in the absence (control) or presence of 100 *μ*M EGTA, or (d) NaCl or KCl medium were (a–d) incubated in the absence (basal) or presence of 575 *μ*M ATP^4−^ (2 mM or 1.4 mM ATP as explained in Section  2.8) at 37°C for (left panels) 15 min or (right panels) 5 min in the presence of 25 *μ*M ethidium^+^. (a–d) Incubations were stopped by the addition of MgCl_2_ medium and centrifugation. Mean fluorescence intensities (MFI) of (left panels) DCF (ROS formation) or (right panels) ethidium^+^ uptake (pore formation) were determined by flow cytometry and results shown as means ± SD, *n* = 3; ***P < 0.001 or **P < 0.01 compared to corresponding basal; ^†††^ *P* < 0.001 compared to corresponding ATP.

P2X7 activation has been reported to induce ROS formation in a number of cell types, including primary microglia [24, 25]. Thus, ATP-induced ROS formation in the EOC13 cell line was investigated using the ROS sensitive probe DCF. Cells loaded with H_2_DCFDA (which is converted to DCF inside cells) were incubated in the absence or presence of ATP, and the subsequent ROS formation analysed by flow cytometry. As extracellular Ca^2+^ has been reported to be important for P2X7-induced ROS formation in a number of cell types [24, 26, 27], assays were initially conducted in the presence of 1 mM Ca^2+^. However, due to the inhibitory action of Ca^2+^ on P2X7 [15], assays were initially conducted with 2 mM ATP. Incubation with 2 mM ATP induced significant ROS formation in EOC13 cells compared to cells incubated in the absence of ATP (MFI of ROS formation 16.3 ± 0.6 and 5.18 ± 0.06, resp., *P* < 0.001, *n* = 3). Furthermore, preincubation of cells with 10 *μ*M AZ10606120 resulted in complete inhibition of ATP-induced ROS formation (Figure 4(a)), indicating that this process is mediated by P2X7 activation. As for cation uptake (Figure 3), AZ10606120 did not significantly alter the basal ROS formation (Figure 4(a)) or cell viability (as assessed by forward and side scatter) (data not shown).

P2X7 is a ligand-gated cation channel [7]; therefore the possible roles for cation fluxes in P2X7-induced ROS formation were next investigated. P2X7-induced ROS formation has been reported to be partially dependent on Ca^2+^ influx in human promyelocytes [26] and rat submandibular gland cells [27]. Thus, to determine if Ca^2+^ influx is required for P2X7-mediated ROS formation in EOC13 cells, ATP-induced ROS formation in the absence and presence of Ca^2+^ was investigated. For this comparison, equivalent amounts of ATP^4−^ (575 *μ*M) were used by adding 1.4 or 2 mM ATP to NaCl medium nominally free of Ca^2+^ or containing 1 mM Ca^2+^, respectively. ATP induced significant ROS formation in both the absence and presence of 1 mM Ca^2+^ compared to similarly treated cells in the absence of ATP (Figure 4(b)). Cells incubated in the absence of Ca^2+^ had significantly higher ATP-induced ROS formation compared to those incubated in the presence of Ca^2+^. In contrast, ATP-induced ethidium^+^ uptake (P2X7 function) was similar in cells incubated in the absence or presence of Ca^2+^ (Figure 4(b)), indicating that the differences in ATP-induced ROS formation were not due to altered P2X7 function.

NaCl medium may contain nominal amounts of Ca^2+^. Thus, to further exclude a role for Ca^2+^ in P2X7-mediated ROS formation in EOC13 cells, ATP-induced ROS formation was investigated in the absence and presence of the Ca^2+^ chelator EGTA. Incubation with 1.4 mM ATP induced significant but similar amounts of ROS formation in both the absence and presence of 100 *μ*M EGTA compared to similarly treated cells in the absence of ATP (Figure 4(c)). Again, ATP-induced ethidium^+^ uptake was similar in cells incubated in the absence or presence of EGTA (Figure 4(c)).

Finally, the role of K^+^ in P2X7-induced ROS formation in EOC13 cells was investigated. Both ROS and K^+^ efflux have been reported to be involved in interleukin-1*β* (IL-1*β*) release from monocytes, although whether these downstream processes are linked is yet to be established [28]. Thus, to determine if K^+^ efflux is involved in P2X7-mediated ROS formation in EOC13 cells, ATP-induced ROS formation was compared with cells in NaCl medium and KCl medium, which prevents the loss of intracellular K^+^. Incubation with 1.4 mM ATP induced significant ROS formation in both NaCl and KCl media, with similar levels of ROS formation in both media (Figure 4(d)). Likewise, ATP-induced ethidium^+^ uptake was similar in NaCl and KCl media (Figure 4(d)), indicating that the inability of high extracellular K^+^ to impair ATP-induced ROS formation was not due to altered P2X7 function.

**Figure 5:**
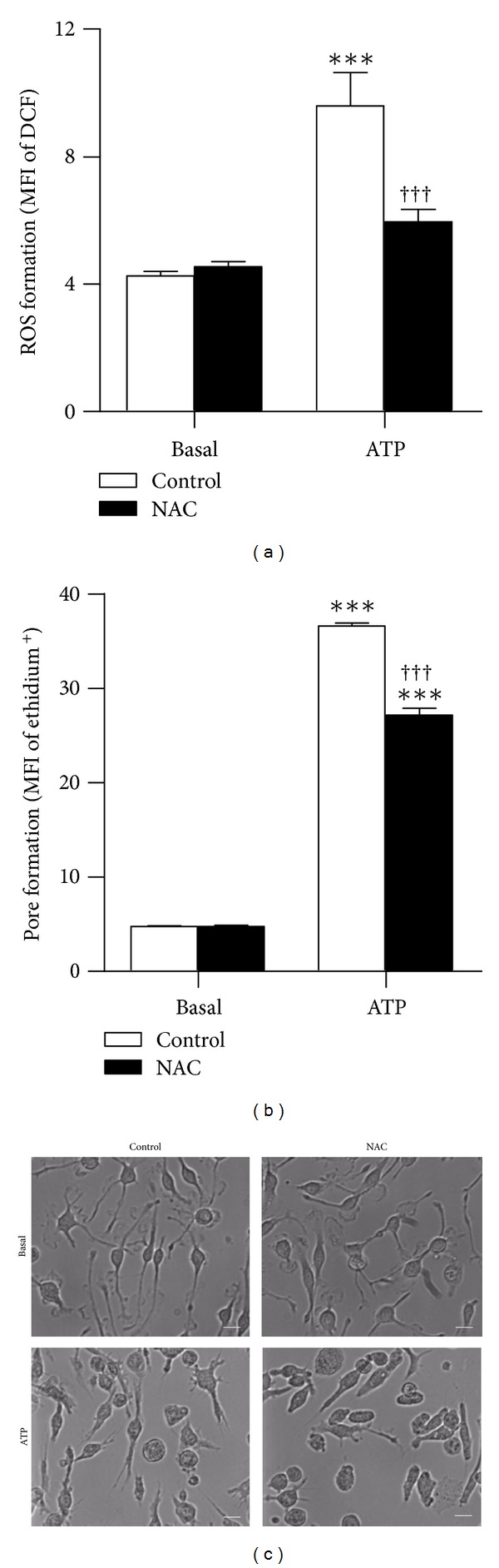
The ROS scavenger NAC inhibits P2X7-induced ROS and pore formation in EOC13 microglial cells. (a and c) Adherent DCF-loaded EOC13 cells or (b) suspended EOC13 cells in NaCl medium were preincubated in the absence (control) or presence of 40 mM NAC at 37°C for 30 min and then in the absence (basal) or presence of 1.4 mM ATP for (a and c) 15 min or (b) 5 min in the presence of 25 *μ*M ethidium^+^. (a–c) Incubations were stopped by the addition of MgCl_2_ medium and (a and b) centrifugation. (a and b) Mean fluorescence intensities (MFI) of (a) DCF (ROS formation) or (b) ethidium^+^ uptake (pore formation) were determined by flow cytometry and results shown as means ± SD, *n* = 3; ***P < 0.001 compared to corresponding basal; ^†††^ *P* < 0.001 compared to corresponding ATP in the absence of NAC. (c) DIC images of cell morphology were acquired by microscopy. Bars represent 20 *μ*m. Results are representative of 2 experiments.

To confirm that P2X7 activation induced ROS formation in EOC13 microglia, DCF-loaded cells in NaCl medium were preincubated in the absence or presence of the ROS scavenger NAC, before incubation in the absence or presence of ATP. As above (Figure 4), 1.4 mM ATP induced significant ROS formation (Figure 5(a)). Preincubation with 40 mM NAC inhibited ATP-induced ROS formation by 73.7 ± 0.3% (Figure 5(a)). Basal ROS formation (Figure 5(a)) and cell viability (as assessed by forward and side scatter) (data not shown) were similar between treatments. Preincubation of cells with 40 mM NAC inhibited ATP-induced ethidium^+^ uptake by 30 ± 2% (Figure 5(b)). Thus, the inhibitory effect of NAC on P2X7-induced ROS formation may be partially attributed to inhibition of P2X7 itself. However, incubation with NAC and ATP, but not either compound alone, reduced the amount of gated viable cells by ~40% in the ethidium^+^ uptake assay (as assessed by forward and side scatter) (data not shown). This suggests that the inhibitory action of NAC on ATP-induced ethidium^+^ uptake may be a result of cytotoxicity in this assay.

To confirm that NAC did not induce morphological changes or cause cytotoxicity under the conditions used for the ROS assay, DIC images of adherent cells were acquired following incubation in the absence or presence of ATP. Cells incubated in the absence or presence of NAC (without ATP) displayed discrete cell bodies with long, spindled shaped processes (Figure 5(c)), as previously observed [13]. Cells incubated in the absence or presence of NAC (with ATP) also displayed discrete cell bodies, but with retracted and branched processes (Figure 5(c)), typical of ATP causing membrane changes [29]. Therefore, in the ROS assay, EOC13 cell morphology was not altered by NAC when compared to the corresponding treatment.

DCF-loaded cells were also preincubated in the absence or presence of the broad-spectrum ROS inhibitor DPI and the ATP-induced ROS formation investigated. However, a 30 min preincubation with DPI at various concentrations (5–40 *μ*M) led to high amounts of cell death (data not shown), and thus this compound was not examined further.

**Figure 6:**
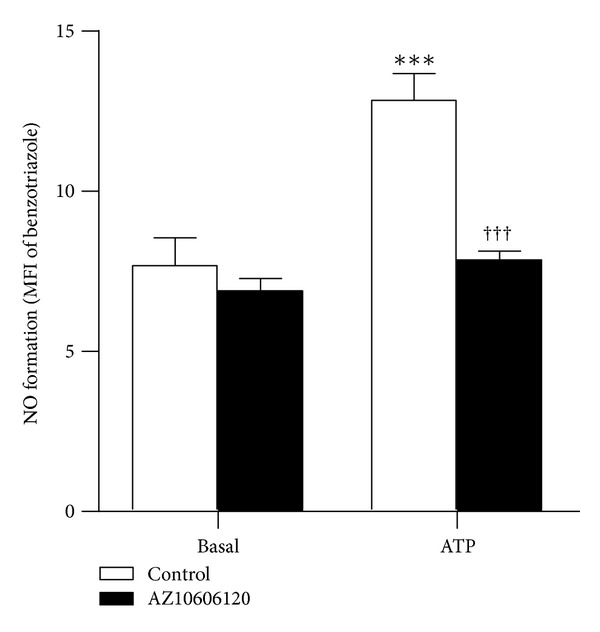
P2X7 activation induces NO formation in EOC13 microglial cells. Adherent DAF-FM DA-loaded EOC13 cells in NaCl medium were preincubated in the absence (control) or presence of 10 *μ*M AZ10606120 at 37°C for 15 min and then in the absence (basal) or presence of 1.4 mM ATP for 15 min. Incubations were stopped by the addition of MgCl_2_ medium and centrifugation. Mean fluorescence intensities (MFI) of benzotriazole (NO formation) were determined by flow cytometry and results shown as means ± SD, *n* = 3; ***P < 0.001 compared to corresponding basal; ^†††^ *P* < 0.001 compared to corresponding ATP.

To further verify that P2X7 activation induces the formation of reactive species in EOC13 cells, ATP-induced NO formation was investigated using the NO sensitive probe DAF-FM DA. Cells loaded with DAF-FM DA (which reacts with NO to form a fluorescent benzotriazole) were preincubated in the absence or presence of AZ10606120, followed by incubation in the absence or presence of ATP, and the subsequent NO formation analysed by flow cytometry. Incubation with 1.4 mM ATP induced significant NO formation in EOC13 cells compared to cells incubated in the absence of ATP (Figure 6). Furthermore, preincubation of cells with 10 *μ*M AZ10606120 inhibited ATP-induced NO formation by 82 ± 11% (Figure 6), indicating that this process is mediated by P2X7 activation. Again, AZ10606120 did not significantly alter the basal NO formation (Figure 6) or cell viability (as assessed by forward and side scatter) (data not shown).

### 3.5. P2X7 Activation Induces Cell Death in EOC13 Microglial Cells

**Figure 7:**
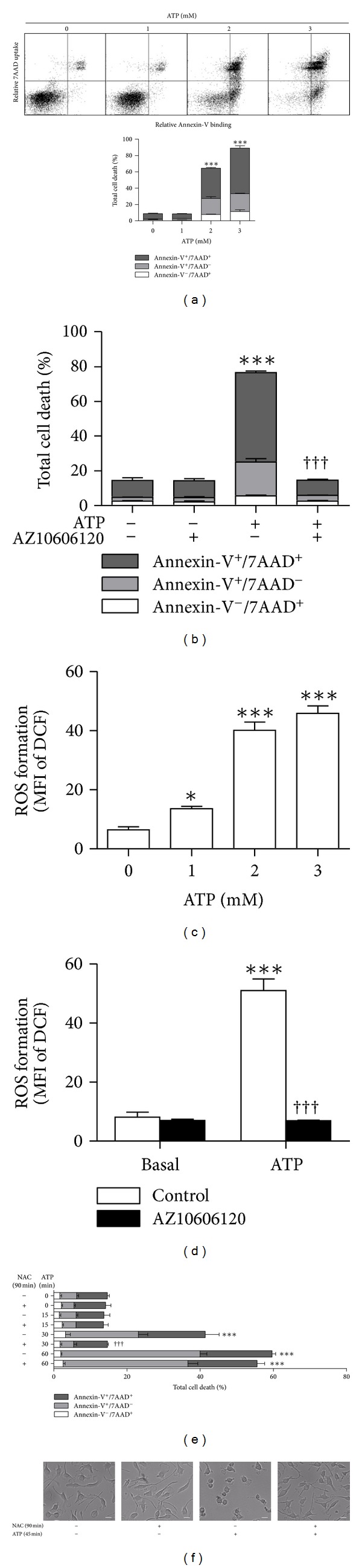
P2X7 activation induces cell death in EOC13 microglial cells. (a) Adherent EOC13 cells in complete DMEM medium were incubated in the absence or presence of varying concentrations of ATP (as indicated) at 37°C for 24 h. (b) Adherent cells in complete DMEM medium were preincubated in the absence or presence of 10 *μ*M AZ10606120 at 37°C for 15 min and then in the absence or presence of 2 mM ATP for 24 h. (e and f) Adherent cells in complete DMEM medium were incubated in the absence or presence of 40 mM NAC at 37°C for 90 min and incubated in the absence or presence of 2 mM ATP for the final (e) 15–60 min or (f) 45 min (of the 90 min incubation), and then the medium replaced with fresh complete DMEM medium for 24 h. (a, b, and e) Cells were harvested, labelled with Annexin-V-Fluorescein and 7AAD, and the percentage of Annexin-V^−^/7AAD^+^, Annexin-V^+^/7AAD^−^, and Annexin-V^+^/7AAD^+^ cells (together representing total cell death) determined by flow cytometry. (f) DIC images of cell morphology were acquired by microscopy. Bars represent 20 *μ*m. (c) Adherent DCF-loaded cells in complete DMEM medium were incubated in the absence (basal) or presence of varying concentrations of ATP (as indicated) at 37°C for 15 min. (d) Adherent DCF-loaded cells in complete DMEM medium were preincubated in the absence (control) or presence of 10 *μ*M AZ10606120 at 37°C for 15 min and then in the absence (basal) or presence of 2 mM ATP for 15 min. (c and d) Incubations were stopped by the addition of MgCl_2_ medium and centrifugation. Mean fluorescence intensity (MFI) of DCF (ROS formation) was determined by flow cytometry. Results shown as (a) dot plots of one representative set of data demonstrating the quadrant markers and (a–e) means ± SD, *n* = 3; ***P < 0.001 or *P < 0.05 compared to (a and c) 0 mM ATP, (b and d) corresponding basal, or (e) corresponding 0 min ATP; ^†††^ *P* < 0.001 compared to corresponding ATP in the absence of (b and d) AZ10606120 or (e) NAC.

P2X7 activation results in the death of various cell types [11, 12]. To determine whether ATP induces the death of EOC13 microglia, cells in complete DMEM medium were incubated in the absence or presence of ATP for 24 h, and then the percentage of Annexin-V^+^/7AAD^−^, Annexin-V^−^/7AAD^+^, and Annexin-V^+^/7AAD^+^ cells examined by flow cytometry (Figure 7(a)). Cell death is expressed as the total of dying (Annexin-V^+^/7AAD^−^) and dead (Annexin-V^−^/7AAD^+^ and Annexin-V^+^/7AAD^+^) cells. Incubation with either 2 or 3 mM ATP but not 1 mM ATP resulted in significantly higher percentages of total cell death compared to cells incubated in the absence of ATP (Figure 7(a)). Next, to determine if the ATP-induced EOC13 death was mediated by P2X7 activation, cells were preincubated in the absence or presence of AZ10606120 and then incubated in the absence or presence of ATP for 24 h. As above (Figure 7(a)), 2 mM ATP induced significant cell death, with higher percentages of total cell death compared to cells incubated in the absence of ATP (Figure 7(b)). Preincubation with 10 *μ*M AZ10606120 completely inhibited ATP-induced cell death (Figure 7(b)), indicating that this process is mediated by P2X7 activation.

P2X7-induced death of murine RAW264.7 macrophages is mediated by ROS formation [30]. Therefore, a role for ROS in P2X7-induced death of EOC13 microglia was investigated. To confirm that P2X7 induced ROS formation under conditions used to induce cell death, DCF-loaded EOC13 cells in complete DMEM medium were incubated in the absence or presence of ATP, and then subsequent ROS formation determined by flow cytometry. Similar to ATP-induced cell death (Figure 7(a)), incubation with 2 or 3 but not 1 mM ATP induced significant ROS formation in EOC13 cells compared to cells incubated in the absence of ATP (Figure 7(c)). The requirement for higher ATP concentrations to induce cell death (Figure 7(a)) or ROS formation (Figure 7(c)) compared to pore formation (Figure 3(b)) is in line with the inhibitory action of divalent cations [15], which are present in the culture medium but not in the NaCl medium used. To confirm that this ROS formation was mediated by P2X7, cells were preincubated with AZ10606120 and the amounts of ATP-induced ROS formation determined. To parallel the cell death experiments, 2 mM ATP was utilised. Again, ATP induced significant ROS formation compared to cells incubated in the absence of ATP (Figure 7(d)). Preincubation with 10 *μ*M AZ10606120 completely inhibited this ATP-induced ROS formation (Figure 7(d)).

Finally, the effect of NAC on P2X7-induced cell death was investigated. EOC13 cells were preincubated in the absence or presence of NAC followed by ATP for 24 h. However, 24 h incubation with 40 mM NAC in the absence of ATP led to significant amounts of EOC13 cell death (data not shown). Therefore, to reduce the total exposure to 40 mM NAC, cells were incubated in the absence or presence of NAC for 90 min, with ATP added in the final 15–60 min. The medium was then replaced with fresh complete DMEM medium and the cells incubated for 24 h. Incubation with 2 mM ATP for 30 or 60 min but not 15 min resulted in significant cell death compared to cells incubated in the absence of ATP (Figure 7(e)). In contrast to the 24 h incubation with NAC, 90 min incubation with 40 mM NAC (without ATP) did not induce significant cell death compared to cells incubated for the same length of time in the absence of both NAC and ATP (Figure 7(e)). A 60 min preincubation with NAC inhibited cell death induced by 30 min incubation with ATP by 99 ± 6% (Figure 7(e)). In contrast, a 75 and 30 min preincubation with NAC, followed by 15 and 60 min ATP treatment, respectively, had no effect on the percentage of cell death compared to cells incubated for the same time length with ATP in the absence of NAC (Figure 7(e)).

To further confirm that NAC did not induce morphological changes or cause cytotoxicity under the conditions used for the cell death assay, DIC images of adherent cells were acquired following the 24 h incubation. As above (Figure 5(c)), cells incubated in the absence or presence of NAC (without ATP) displayed discrete cell bodies with long, spindled shaped processes (Figure 7(f)). In addition, cells incubated in the presence of NAC and ATP displayed a similar morphology to that of cells incubated in the absence of ATP (Figure 7(f)). In contrast, cells preincubated with ATP alone displayed rounded and granular cell bodies with no or few processes (Figure 7(f)), characteristic of cell death. Furthermore, wells preincubated with ATP alone had a high amount of nonadherent cells compared to the other treatments (data not shown). Thus, preincubation with NAC prevented the morphological changes associated with ATP incubation, but NAC alone had no effect on cell morphology.

## 4. Discussion

The current study demonstrates for the first time that the murine microglial EOC13 cell line expresses functional P2X7. Firstly, the presence of P2X7 mRNA and protein was established using RT-PCR and immunoblotting techniques. In addition, the presence of cell-surface P2X7 was demonstrated using immunofluorescence staining. Moreover, P2X7 on EOC13 microglia was functional, as the P2X7 agonists ATP and BzATP induced significant ethidium^+^ or YO-PRO-1^2+^ uptake into these cells. In these experiments, ATP induced ethidium^+^ uptake with an EC_50_ value which falls within the typical range for ATP-induced cation fluxes mediated by recombinant murine P2X7 [31]. Furthermore, pretreatment of cells with P2X7 antagonists inhibited ATP-induced organic cation uptake. Lastly, ATP could induce ROS formation in and the death of EOC13 cells, and both of these events, which are often associated with P2X7 activation [10–12], could be inhibited by the P2X7 antagonist AZ10606120. The presence of functional P2X7 on EOC13 microglia is consistent with the presence of this receptor on primary microglia and microglial cell lines (including N9, N13, BV-2, and NTW8 cells) [32–35].

P2X7 activation induced ROS formation in EOC13 microglia. P2X7-induced ROS formation has been reported in primary microglia [24, 25], and a role for this process in microglia has been highlighted by several studies. P2X7 activation induces the production of the ROS, superoxide, in primary rat microglia, while this receptor is upregulated in a transgenic mouse model of Alzheimer's disease [25]. Although a direct link between P2X7, superoxide, and Alzheimer's disease was not established, the authors proposed a link between these molecules and this disease. This link is supported by subsequent observations by others, where fibrillar *β*-amyloid peptide, which is associated with Alzheimer's disease, caused ATP release and autocrine activation of P2X7 leading to ROS formation in primary rat microglia [24]. In addition, another group demonstrated that P2X7-induced superoxide release from primary rat microglia induced injury of rat cortical neurons [36]. Collectively, this data indicates that P2X7-induced ROS formation from microglia may be involved in various neuroinflammatory and neurodegenerative disorders. This may be of particular importance in diseases where microglial P2X7 is reported to be upregulated such as in Alzheimer's disease, multiple sclerosis, and amyotrophic lateral sclerosis [25, 37]. It should be noted, however, that DCF, as employed in the current study and as widely used by others to detect ROS, can also propagate ROS formation [38]. Nevertheless, our observation that P2X7 activation also induces the formation of NO in EOC13 cells supports a role for this receptor in the formation of reactive species.

The current study excluded an essential role for Ca^2+^ influx in P2X7-induced ROS formation in EOC13 microglia. This finding is similar to other observations with other murine cell types, including submandibular glands [39] and erythroid cells [40]. In contrast, P2X7-induced ROS formation in primary rat microglia [24] and rat submandibular glands [27] is dependent on an influx of Ca^2+^. The reason for this difference between these two species remains unknown but may reflect differences in experimental protocols or differences in signalling molecules between mice and rats. The current study also excluded an essential role for K^+^ efflux in P2X7-induced ROS formation in EOC13 microglia. Both ROS formation and K^+^ efflux are involved in IL-1*β* release from monocytes, although whether these downstream processes are linked has not been established [28]. Thus, our results indicate that P2X7-induced ROS formation does not require K^+^ efflux and that ROS formation and K^+^ efflux may be independent events in IL-1*β* release from myeloid cells.

P2X7 activation also induced cell death in EOC13 microglia. Use of an Annexin-V/7AAD assay suggested that this process was mediated by apoptosis. However, in the absence of other markers of apoptosis and necrosis, this remains to be established, especially since P2X7 activation induces both apoptosis and necrosis in the microglial N13 cell line [41]. Nevertheless, our observations support previous studies in which P2X7 activation induced death in primary microglia and other microglial cell lines [41, 42]. The physiological role of P2X7-induced microglia death is unclear. Further obscuring this is the known role of P2X7 activation in inducing the proliferation of microglia [43]. This paradoxical role of P2X7 is thought to be related to the relative ATP concentration, with high concentrations promoting cell death and low concentrations promoting cell proliferation [44]. In support of this, our study observed that ATP only induced EOC13 cell death at 2 or 3 but not 1 mM ATP. Moreover, our data also showed that a transient incubation with ATP of 30–60 but not 15 min induced cell death in EOC13 microglia. This suggests that transient ATP release and subsequent P2X7 activation may be sufficient to kill microglia *in vivo*.

The current study examined a potential link between P2X7-induced ROS formation and death in EOC13 microglia. A previous study demonstrated that the ATP-induced death of murine RAW264.7 macrophages was mediated by ROS derived from NADPH oxidase downstream of P2X7 activation [30]. This contrasts with another study, which found that P2X7-induced ROS formation, but not death, was attenuated in primary macrophages from NADPH oxidase deficient mice [45]. Our data using the ROS scavenger NAC supports a role for ROS formation in the P2X7-induced death of EOC13 microglia. The capacity of NAC to prevent P2X7-induced EOC13 microglia death was dependent on the preincubation time with NAC, as well as the total incubation time with ATP, with only 45–60 min preincubations with NAC preventing cell death induced by transient 30–45 min exposures to ATP. In contrast, 24 h incubation with NAC induced significant amounts of EOC13 microglia death, equivalent to that induced by ATP alone. This cytotoxicity of NAC may have occurred due to increased toxic metabolic byproducts such as reduced glutathione [46]. Alternatively, scavenging of ROS by NAC may indicate that low amounts of ROS are important for EOC13 cell homeostasis. Of note, the ROS inhibitor DPI also induced the death of EOC13 microglia, albeit over a much faster time course. Finally, it should be noted that NAC inhibition of P2X7-induced death and ROS formation in EOC13 microglia may have been partly due to direct inhibition of P2X7. NAC inhibited ATP-induced pore formation by 30% compared to a 74 and 99% inhibition of ATP-induced ROS formation and cell death, respectively. This direct inhibition of P2X7 by NAC was not due to an acidic pH, which is known to impair P2X7 function [47], as the NAC-containing solutions were adjusted to pH 7.4 before each assay. Thus, our results indicate that either cellular signalling involving ROS may modulate P2X7 activation in EOC13 microglia or that NAC may directly impair P2X7 at 40 mM. The concentration of NAC used in these experiments (40 mM) is 4–8-fold higher than that used in a number of similar studies (e.g., [48]). The requirement for this high concentration of NAC remains unknown but may reflect a reduced ability of NAC to cross the plasma membrane or to be converted to glutathione in EOC13 cells.

The presence of functional P2X7 on J774 macrophage cells was confirmed in the current study. P2X7 is present in this cell line [17], and activation of P2X7 leads to the release of mature IL-1*β* [49], the formation of macrophage-derived multinucleated giant cells [50–52], and cell death [53]. In this study, the potency of four P2X7 antagonists against 1 mM ATP, the ATP concentration most commonly used to study P2X7, was determined. The IC_50_ values for BBG, A438079, and AZ11645373 (1.8, 7.9, and 1.5 *μ*M, resp.) were within one log range of those published for recombinant murine P2X7 [54, 55]. In contrast, the IC_50_ value for AZ10606120 has not been reported for murine P2X7, although this compound has been shown to specifically bind to and inhibit rat and human P2X7 [20]. In the current study, this highly specific P2X7 antagonist also completely impaired ATP-induced ethidium^+^ uptake, ROS formation, and death of murine EOC13 cells. Thus, AZ10606120 will be useful for future studies of murine P2X7.

In the CNS, extracellular ATP acting through P2X7 on microglia is an important mediator of neuroinflammation [9]. ATP acts as a neurotransmitter and is released from neurons during synaptic transmission and from dying cells [56]. Under normal physiological conditions, extracellular ATP concentrations in the CNS are estimated to be in the nanomolar to micromolar range, depending on the balance between ATP release and degradation, while intracellular microglial ATP concentrations are in the millimolar range [57]. After CNS injury, however, extracellular ATP concentrations increase and can reach as high as the millimolar range [57]. Furthermore, it is hypothesised that ATP may act on microglial P2X7 at very close range where the concentration of ATP may be quite high. Activation of P2X7 on primary microglia and microglial cell lines leads to the release of proinflammatory IL-1*β* and tumour necrosis factor-*α* [33, 58] and ROS formation [24]. Although proinflammatory factors are important for immunity [1], prolonged or inappropriate release of such factors from chronically activated microglial can be highly toxic to neurons and can promote neuroinflammation and neurodegeneration [5]. There are a number of diseases in the CNS characterised by the presence of activated microglia, including Alzheimer's disease, prion infection, cerebral ischemia, multiple sclerosis, and amyotrophic lateral sclerosis. In such diseases, P2X7 has also been reported to be upregulated [25, 37, 59, 60]. This raises questions of possible roles for P2X7 in mediating inappropriate microglial responses in CNS disorders.

## 5. Conclusions

This study demonstrates that EOC13 microglial cells express functional P2X7. Activation of this receptor by ATP resulted in organic cation uptake, ROS formation, and death in these cells. Moreover, the EOC13 cell line may be useful for investigating P2X7-mediated events in microglia and the role of this receptor in microglia-mediated inflammatory disorders.

## Abbreviations

7AAD: 7-Aminoactinomycin D
Ab: Polyclonal antibody
APC: Allophycocyanin
ATP: Adenosine-5′-triphosphate
BBG: Brilliant Blue G
BzATP: 2′(3′)-O-(4-Benzoylbenzoyl) ATP
CNS: Central nervous system
DAF-FM DA: 2′,7′-Difluorofluorescein diacetate
DCF: Dichlorofluorescein
DIC: Differential interference contrast
DMSO: Dimethyl sulfoxide
DPI: Diphenyleneiodonium
EC_50_: Half maximal effective concentration
EGTA: Ethylene glycol tetraacetic acid
FBS: Fetal bovine serum
H_2_DCFDA: 2′,7′-Dichlorodihydrofluorescein diacetate
IC_50_: Half maximal inhibitory concentration
IL-1*β*: Interleukin-1*β*
mAb: Monoclonal antibody
MFI: Mean fluorescence intensity
NAC: N-Acetyl-L-cysteine
NHS: Normal horse serum
NO: Nitric oxide
PBS: Phosphate-buffered saline
PMSF: Phenyl-methyl-sulfonyl-fluoride
ROS: Reactive oxygen species.
